# Visual Perceptual Load Attenuates Age-Related Audiovisual Integration in an Audiovisual Discrimination Task

**DOI:** 10.3389/fpsyg.2021.740221

**Published:** 2021-09-29

**Authors:** Yanna Ren, Hannan Li, Yan Li, Tao Wang, Weiping Yang

**Affiliations:** ^1^Department of Psychology, College of Humanities and Management, Guizhou University of Traditional Chinese Medicine, Guiyang, China; ^2^University Science Park Management Center, Guiyang University, Guiyang, China; ^3^Department of Light and Chemical Engineering, Guizhou Light Industry Technical College, Guiyang, China; ^4^Department of Psychology, Faculty of Education, Hubei University, Wuhan, China

**Keywords:** audiovisual integration, perceptual load, older adults, race model, discrimination task

## Abstract

Previous studies confirmed that the cognitive resources are limited for each person, and perceptual load affects the detection of stimulus greatly; however, how the visual perceptual load influences audiovisual integration (AVI) is still unclear. Here, 20 older and 20 younger adults were recruited to perform an auditory/visual discrimination task under various visual perceptual-load conditions. The analysis for the response times revealed a significantly faster response to the audiovisual stimulus than to the visual stimulus or auditory stimulus (all *p* < 0.001), and a significantly slower response by the older adults than by the younger adults to all targets (all *p* ≤ 0.024). The race-model analysis revealed a higher AV facilitation effect for older (12.54%) than for younger (7.08%) adults under low visual perceptual-load conditions; however, no obvious difference was found between younger (2.92%) and older (3.06%) adults under medium visual perceptual-load conditions. Only the AV depression effect was found for both younger and older adults under high visual perceptual-load conditions. Additionally, the peak latencies of AVI were significantly delayed in older adults under all visual perceptual-load conditions. These results suggested that visual perceptual load decreased AVI (i.e., depression effects), and the AVI effect was increased but delayed for older adults.

## Introduction

Individuals simultaneously receive many types of sensory information from the outside world, including visual, auditory, tactile, olfactory, and gustatory information; however, our brain can select and merge the available information to facilitate the perception of the outside world. For example, when driving a car, it is necessary to watch the road condition (visual information), listen to the voice broadcast (auditory information), and operate the steering wheel (tactile information) simultaneously. The interactive processing of information from multiple sensory modalities is called multisensory integration. Individuals acquire most of the information from the environment through visual and auditory modalities, and numerous studies have found that responses to audiovisual stimulus were faster and more accurate than those to auditory-only or visual-only stimulus (Meredith et al., 1987; Stein and Meredith, 1993; Stein, 2012). The procedure that merges visual and auditory information is defined as audiovisual integration (AVI), which assists individuals in identifying objects much more easily and is the topical issue of multisensory integration (Raab, 1962; Miller, 1982). Ordinary life is complex and AVI might be disturbed by various distractors from the surrounding environment. Furthermore, for each person, the cognitive resources are limited, that is, if the cognitive demand is higher for one task, less will be left to process other tasks, known as “perceptual load theory” (Kahneman, 1973; Lavie and Tsal, 1994; Lavie, 1995). Under different perceptual loads, how individuals efficiently integrate visual and auditory information has been a hot multisensory research topic in recent years (Alsius et al., 2005, 2007; Baseler et al., 2014; Wahn and König, 2015; Ren et al., 2020a).

Alsius et al. (2005, 2014) and Ren et al. (2020a) investigated the additional perceptual load of visual transients on AVI. In studies by Alsius et al. (2005, 2014) McGurk stimuli were used in audiovisual redundant tasks to assess AVI, and meaningless checkerboard images and white noise were used in the study by Ren et al. (2020a). In the low perceptual-load condition, participants were instructed to respond only to the audiovisual redundant task but to respond to the visual distractor at the same time in the high perceptual-load condition. Consistent results showed that the AVI was lower in the condition with high perceptual load of visual transients (dual task) than in the condition with a low perceptual load of visual transients (single task). In the studies by Alsius et al. (2005, 2014) and Ren et al. (2020a), the visual distractors were transitorily presented and accompanied by stimuli in an audiovisual redundant task. To investigate the influence of sustained visual distractors on AVI, Wahn and König (2015) instructed participants to continuously track visual moving balls when performing the audiovisual redundancy task, and their results revealed that AVI was comparable under high sustained visual perceptual-load conditions and low sustained visual perceptual-load conditions, indicating that sustained visual perceptual load did not disrupt AVI. Dual tasks were employed in the abovementioned studies, in which one task was used to assess AVI and the other was used to manipulate visual perceptual load, and the visual perceptual load was from the additional distractor, during which the low perceptual-load condition involved focused attention but high perceptual-load condition involved divided attention. Therefore, it is difficult to evidence whether the perceptual load influences AVI or attention clearly. To clarify this question, the current study aimed to investigate how the visual perceptual load (demand) from the visual stimulus itself influences AVI in a single task.

Macdonald and Lavie (2011) found that visual perceptual load severely affected the auditory perception and even induced “inattentional deafness,” showing that the participant failed to notice the tone (∼79%) during the visual detection task under high visual perceptual load (Macdonald and Lavie, 2011). Diederich et al. (2008) proposed that AVI occurred only when the processing of auditory and visual information was completed in a specific time frame. Considering the limitation of nerve-center energy proposed by Kahneman (1973) in “Attention and Effort,” with increasing visual perceptual load, more perceptual resources occupied by visual stimuli and the processing of auditory stimuli will undoubtedly be limited. Colonius and Diederich (2004) reported that the AVI effect was greater when the intensities of visual and auditory stimuli were equalized, and it is relatively lower if they were not coordinated (Colonius and Diederich, 2004). Therefore, we hypothesized that the AVI effect is higher for equalized pairs (low visual perceptual-load conditions) than for incoordinate pairs (high visual perceptual-load conditions). The hypothesis was tested using a visual/auditory discrimination task with different visual perceptual-load stimuli. If the AVI was lower under the higher visual perceptual-load conditions than under the low or medium visual perceptual-load conditions, it would be concluded that the visual perceptual load greatly affected the AVI and that the visual perceptual load reduced the AVI effect.

Additionally, aging is an important factor that influences AVI, and some researchers reported that the AVI was higher for older adults than for younger adults (Laurienti et al., 2006; Peiffer et al., 2007; Stephen et al., 2010; Ren et al., 2020b), but how AVI changed with the alteration of visual perceptual load for older adults is also still unclear. Recently, some of them proposed that higher AVI in older adults is an adaptive mechanism to compensate for unimodal sensory decline (Laurienti et al., 2006; Peiffer et al., 2007; Freiherr et al., 2013; Ren et al., 2020c), and the audiovisual perceptual training could improve cognitive function of healthy older adults (Anguera et al., 2013; Yang et al., 2018; O’Brien et al., 2020) and patients with mild cognitive impairment (Lee et al., 2020). Therefore, it is necessary to investigate the effect of the perceptual load of visual stimuli on AVI to clarify the interaction of perceptual load and AVI, which might further offer a reference for the selection of experimental materials in cognitive training tasks. Therefore, another aim of the present study was to investigate the aging effect on the interaction between AVI and visual perceptual load. Considering that auditory and visual stimulus processing is generally slower in older adults (Grady, 2008; Anderson, 2019), but they are still able to correctly perceive the outside world, we hypothesized that a compensatory mechanism also existed for older adults during AVI even under visual perceptual-load conditions. However, one of the well-documented effects of aging is that there is a reduction in the total amount of resources available for information processing (Cabeza et al., 2006; Paige and Gutchess, 2015); thus, performance might be even worse in high visual perceptual-load condition. In the current study, the hypothesis was tested by comparing AVI between older and younger adults under all visual perceptual-load conditions. If the AVI effect of the older adults is higher than that of younger adults under any visual perceptual-load conditions, it would be concluded that the older adults might establish a compensatory mechanism.

## Materials and Methods

### Subjects

The sample size was calculated using the G^∗^Power 3.1.9.2 program.^1^ The total sample size was 36, with an effect size *f* of 0.4 and a power (1-β err prob) of 0.8 (Lakens, 2021). Therefore, 20 healthy older adults (59–73 years, mean age ± SD, 63 ± 4) and 20 younger college students (20–23 years, mean age ± SD, 22 ± 1) were recruited to participate in this study. All older adults were recruited from Guiyang City, and all younger adults were undergraduates. All participants had normal hearing, had a normal or corrected-to-normal vision, had no color blindness or color weakness, and were not informed of the aim of this study. Those participants whose mini-mental state examination (MMSE) scores were out of the range of 25–30 were excluded from the experiment (Bleecker et al., 1988). All the participants signed the informed consent form approved by the Ethics Committee of the Second Affiliated Hospital of Guizhou University of Traditional Chinese Medicine, and obtained the remuneration for their time.

### Stimuli

**FIGURE 1:**
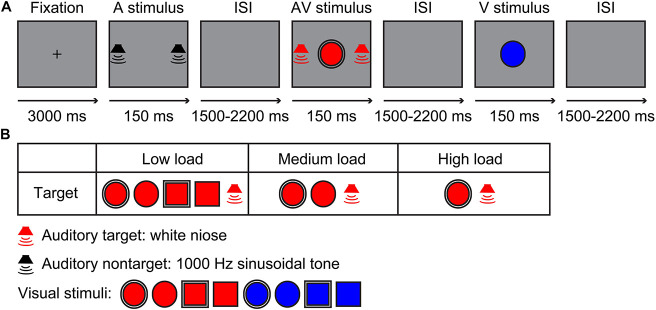
Experimental design. **(A)** A possible sequence for the auditory non-target, audiovisual target, and visual non-target stimuli in the experiment. **(B)** Stimuli types. Low load, low visual perceptual-load condition; Medium load, medium visual perceptual-load condition; High load, high visual perceptual-load condition.

The visual stimuli included a red/blue circle and square with a 2.6-cm diameter single- or dual-border (Bruner et al., 1956; Figure 1B). The auditory stimuli contained a 60-dB white noise for auditory target stimuli and 60-dB 1,000-Hz sinusoidal tone for auditory non-target stimuli, which were edited using Audacity 2.3.0^2^ for a duration of 150 ms (10 ms of rise or/fall cosine gate) (Ren et al., 2016, 2018). The experiment contained three sessions, namely, a low visual perceptual-load session, a medium visual perceptual-load session, and a high visual perceptual-load session. For low visual perceptual-load session, the visual target stimuli were red circles and squares with single- or dual-border, and the visual non-target stimuli were blue circles and squares with single- or dual-border (Figure 1B). For medium visual perceptual-load session, the visual target stimuli were red circles with single or dual borders, and the visual non-target stimuli included red squares with single or dual borders and blue circles and squares with single or dual borders (Figure 1B). For high visual perceptual-load session, the visual target stimuli were red circles with dual borders, and the visual non-target stimuli included red circles with single borders, red squares with single or dual borders, and blue circles and squares with single or dual borders (Figure 1B). Under all of the visual perceptual-load conditions, the audiovisual target stimuli were the simultaneous presentation of the auditory target stimulus and visual target stimulus, and the audiovisual non-target stimulus was the simultaneous presentation of the auditory non-target stimulus and visual non-target stimulus. The following two conditions: the visual non-target stimulus accompanied by auditory target stimulus and the visual target stimulus accompanied by auditory non-target stimulus were not included.

### Procedure

The experiment was conducted in a dimly lit and sound-attenuated room (Cognitive Psychology Laboratory, Guizhou University of Traditional Chinese Medicine, China). The presentation for all stimuli and collection for behavioral response were controlled by E-prime 2.0 (Psychology Software Tools, Inc., Pittsburgh, PA, United States). The visual stimulus (V) was presented on the screen of the computer in front of the participant with 60-cm distance, and the background of the monitor (Dell, E2213c) was gray during the experiment. The auditory stimulus (A) was presented through two speakers (Edifier, R19U) located on the right and the left of the screen. Each session started with a 3,000-ms fixation, and then, all stimuli (A,V,AV) were presented for 150 ms randomly with a randomized interstimulus interval (ISI) of 1,500–2,200 ms (Figure 1A). The participant was instructed only to respond to target stimuli by pressing the right button of the mouse as accurately and rapidly as possible, but withhold responses for non-target stimuli. In total, three sessions were conducted randomly, and each session lasted for 10 min with suitable rest according to the physical condition of the participant individually. Each session contained 240 trials, including 120 target and 120 non-target stimuli, with 40 trials for each stimulus type.

### Data Analysis

The accuracy of responding is the percentage of correct responses (the response time falling within the average time duration ± 2.5 SD) relative to the total number of target stimuli. The response times (RTs) and accuracy for each participant under each condition were calculated separately, and then, submitted to a 2_group_ (older, younger) × 3_load_ (low, medium, high) × 3_stimulus_ (A,V,AV) ANOVA with Greenhouse–Geisser corrections followed by a *post hoc* analysis.

**FIGURE 2:**
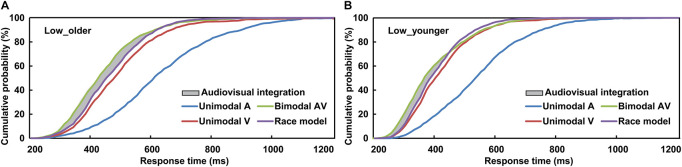
Cumulative distribution functions of auditory stimuli, visual stimuli, race model, and audiovisual stimuli for older **(A)** and younger **(B)** adults under low visual perceptual-load conditions. Low_older, low visual perceptual-load condition for older adults; Low_younger, low visual perceptual-load condition for younger adults.

To interpret the phenomenon that the response to AV stimulus was obviously faster than to A or V stimulus, the separate-activation model and coactivation model were raised (Meredith et al., 1987; Stein and Meredith, 1993; Stein, 2012). The separate-activation model is that the processing of auditory and visual information never combined, and the response was induced by the winner of the race, and so it was also called “race model.” However, the coactivation model is that the activation of auditory and visual was combined and this induced the response cooperatively. The race model was calculated based on the cumulative distribution functions (CDFs) of visual-only and auditory-only response in 10-ms time bins, P(RM) = [P(V) + P(A)]-P(V) × P(A) (Miller, 1982, 1986; Laurienti et al., 2006). P(A) and P(V) were the response probability to a visual-only or auditory-only trial within a given timeframe, respectively, and P(RM) is the predicted response probability for AV trial basing on P(A) and P(V) (Figures 2A,B). If P(AV) is significantly different from P(RM), which suggests the AV violated the race model and the co-activation model was applied, then the AVI was assumed to have occurred (*t-test, p* ≤ 0.05). If P(AV) was significantly greater than P(RM), it was defined as AV facilitation, otherwise, AV depression (Meredith et al., 1987; Stein and Meredith, 1993). As previously studied, the AVI effect was assessed using race model by analyzing the RTs data (Laurienti et al., 2006; Peiffer et al., 2007; Stevenson et al., 2014; Ren et al., 2016, 2018). A probability-difference curve was generated by subtracting the P(RM) of an individual from his/her audiovisual CDFs [P(AV)] in each 10-ms time bin, and the peak value (peak benefit) of the curve was used to assess AVI ability (Xu et al., 2020). The time frame from the stimulus onset to peak benefit was peak latency, which is an important index to evaluate when AVI occurred (Laurienti et al., 2006; Peiffer et al., 2007; Ren et al., 2016, 2018). SPSS version 19.0 software (SPSS, Tokyo, Japan) was used for all statistical analyses.

## Results

### Accuracy

**FIGURE 3:**
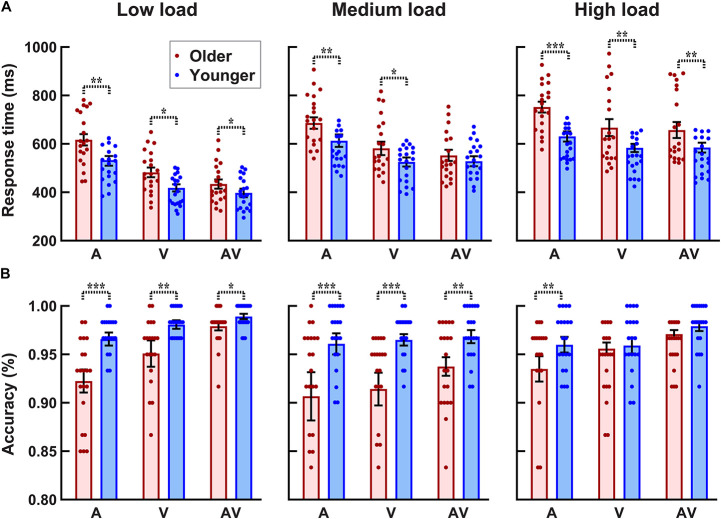
Higher response time **(A)** and lower accuracy **(B)** for older adults than for younger adults under all visual perceptual-load conditions. *p < 0.05, **p < 0.01, ***p < 0.001. Low load, low visual perceptual-load condition; Medium load, medium visual perceptual-load condition; High load, high visual perceptual-load condition.

As shown in Figure 3B, the accuracy for each participant and each condition was higher than 80%. The 2_group_ (older, younger) × 3_load_ (low, medium, high) × 3_stimulus_ (A,V,AV) ANOVA analysis revealed a significant group main effect [*F*(1, 38) = 9.289, *p* = 0.004, η*_*p*_*^2^ = 0.196], showing that the accuracy was lower for older adults than that for younger adults. There was a significant perceptual load main effect [*F*(2, 76) = 7.026, *p* = 0.003, η*_*p*_*^2^ = 0.156], showing that the accuracy under low and medium visual perceptual-load conditions was higher than that under high visual perceptual-load conditions. Additionally, a significant stimulus type main effect was also found [*F*(2, 76) = 13.038, *p* < 0.001, η*_*p*_*^2^ = 0.255], showing that the accuracy was higher when responding to bimodal AV stimulus than unimodal V or A stimulus (AV > V > A). In addition, the interaction of perceptual load × group was marginally significant [*F*(2, 76) = 3.368, *p* = 0.049, η*_*p*_*^2^ = 0.081]. The *post hoc* analysis using pairwise comparison with Bonferroni correction for perceptual load revealed that the accuracy was lower under high visual perceptual-load conditions than that under low (*p* = 0.004) and medium (*p* = 0.018) visual perceptual-load conditions, but there was no significant difference between the low and medium visual perceptual-load conditions (*p* = 1.000) for older adults. The *post hoc* analysis using pairwise comparison with Bonferroni correction for the group revealed that the accuracy for older adults was lower than that for younger adults under both low (*p* = 0.003) and medium (*p* = 0.009) visual perceptual-load conditions, but no significant difference was found between older and younger adults (*p* = 0.168) under high visual perceptual-load conditions.

### Response Times

The 2_group_ (older, younger) × 3_load_ (low, medium, high) × 3_stimulus_ (A,V,AV) ANOVA analysis for RTs (Figure 3A) revealed significant group main effect [*F*(1, 38) = 5.528, *p* = 0.024, η*_*p*_*^2^ = 0.127], showing that the response was slower by older adults than that by younger adults. There were significant perceptual load main effects [*F*(2, 76) = 166.072, *p* < 0.001, η*_*p*_*^2^ = 0.814], showing that the response under the low visual perceptual-load conditions was fastest (low > medium > high). Additionally, significant stimulus type main effect was also found [*F*(2, 76) = 105.521, *p* < 0.001, η*_*p*_*^2^ = 0.156], showing that the response to AV stimulus was faster than that to A or V stimulus (AV > V > A). The interaction of perceptual load × group was marginally significant [*F*(2, 76) = 3.324, *p* = 0.048, η*_*p*_*^2^ = 0.080]. The *post hoc* analysis using pairwise comparison with Bonferroni correction revealed that the response by older adults was obviously slower than that by younger adults under low (*p* = 0.028) and high (*p* = 0.009) visual perceptual-load conditions, but no significant difference was found between younger and older adults under medium visual perceptual-load conditions (*p* = 0.125). Additionally, under all perceptual-load conditions, the response was slower by older adults than that by younger adults (all *p* ≥ 0.002). The interaction of perceptual load × stimulus was also significant [*F*(4, 152) = 18.927, *p* < 0.001, η*_*p*_*^2^ = 0.332]. The *post hoc* analysis using pairwise comparison with Bonferroni correction for perceptual load revealed that under low and medium visual perceptual-load conditions, the response to the three stimuli were significantly different (AV > V > A, all *p* ≤ 0.004). However, under the high visual perceptual-load conditions, the response to A stimulus was slower than that to AV (*p* < 0.001) or V (*p* < 0.001) stimulus, but no significant difference was found between AV and V stimuli (*p* = 1.000). Additionally, the response among all visual perceptual-load conditions was significantly different for all stimuli (low > medium > high, all *p* < 0.001).

### Race Model Analysis

**FIGURE 4:**
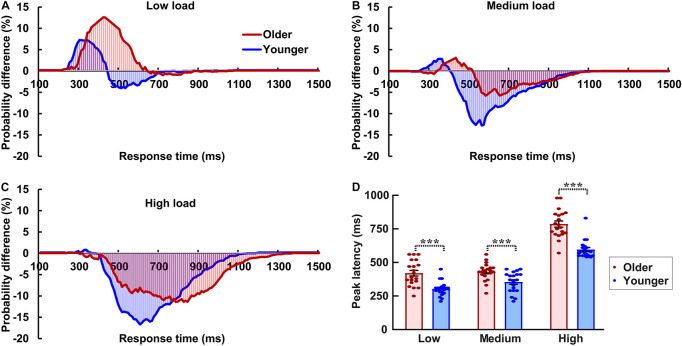
Significant audiovisual facilitation effect was mainly found in the low visual perceptual-load conditions **(A)**, but depression effects were found in the medium **(B)** and high **(C)** visual perceptual-load conditions. The AVI was delayed for older adults than that for younger adults under all visual perceptual-load conditions. **(D)** Low, low visual perceptual-load condition; Medium, medium visual perceptual-load condition; High, high visual perceptual-load condition. ***p < 0.001.

**FIGURE 5:**
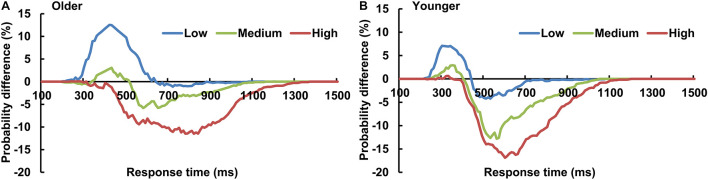
The AVI effect changed from facilitation to depression for both older **(A)** and younger **(B)** adults with increasing of visual perceptual load. Low, low visual perceptual-load condition; Medium, medium visual perceptual-load condition; High, high visual perceptual-load condition.

The AVI was assessed using race model under all visual perceptual-load conditions, as Figure 2A for older adults and Figure 2B for younger adults under low perceptual-load conditions. Significant AVI was found under all visual perceptual-load conditions for both older (two-tailed *t*-test, all *p* ≤ 0.047) and younger (two-tailed *t*-test, all *p* ≤ 0.05) adults. The AV facilitation effect for older adults (12.54%) was higher than that for younger adults (7.08%) under low visual perceptual-load conditions (Figure 4A), but under medium visual perceptual-load conditions no significant difference was found between older (3.06%) and younger adults (2.92%) (Figure 4B). Under high visual perceptual-load conditions, only the AV depression effect was found in both older (−11.47%) and younger adults (−16.89%) (Figure 4C). These results indicated the AVI was higher in older adults, and the facilitation effect was absent and even the depression effect occurred with an increase in the visual perceptual load for both older (Figure 5A) and younger (Figure 5B) adults. Additionally, the peak latencies were delayed for older adults under all visual perceptual-load conditions than that for younger adults, exhibiting 420 vs. 300 ms, 430 vs. 350 ms, and 850 vs. 600 ms for low, medium, and high visual perceptual-load conditions, respectively (Figure 4D), indicating delayed AVI for older adults than that for younger adults.

## Discussion

The current study aims to investigate the influence of visual perceptual load on AVI and its aging effect. The results showed that the AV facilitation effect was higher for older adults than that for younger adults under low visual perceptual-load conditions, and no obvious difference was found between younger and older adults under medium visual perceptual-load conditions; however, only the AV depression effect was found for both younger and older adults under high visual perceptual-load conditions. Additionally, the AVI was delayed in older adults compared with younger adults under all visual perceptual-load conditions.

Unexpectedly, the AVI effect was altered from facilitation to depression with the increasing visual perceptual load. According to the nerve-center energy theory proposed by Kahneman (1973)in “Attention and Effort” (1973), the complex stimulus can expend more energy and the simple stimulus can expend less energy. The visual task is a simple “feature identification” (single feature) under low visual perceptual-load conditions, but it is complex “object identification” (conjunction of features) under medium (two features) and high (three features) visual perceptual-load conditions. Therefore, more energy was allocated to the visual stimulus under medium visual perceptual-load conditions than that under low visual perceptual-load conditions, and more under high visual perceptual-load conditions than that under medium visual perceptual-load conditions. Additionally, a recent study reported that AVI was sensitive to the amount of available cognitive resources (Michail et al., 2021). In the current study, the memory loads were different among perceptual-load conditions, showing the highest memory load under high perceptual-load conditions (three attributes) and the lowest memory load under low perceptual-load conditions (one attribute). Therefore, more cognitive resources are allocated to the visual stimulus under medium visual perceptual-load conditions than that under low visual perceptual-load conditions, and more under high visual perceptual-load conditions than that under medium visual perceptual-load conditions. The multisensory integration occurred in the human primary visual cortex (Murray et al., 2016). The increased visual demands could, theoretically, limit resources for integrative processing. With increasing perceptual load, more resources were shifted to visual stimuli, which reduced auditory weighting, and therefore limited the benefits of integration (Deloss et al., 2013). Therefore, it is reasonable that the AV facilitation effect was mainly found under low visual perceptual-load conditions and it decreased with the increasing visual perceptual load. However, there is a limitation that it was impossible to rule out of the role of memory load, and precise experimental designs are necessary for future studies. The biased competition model assumes that nerve-center energy enhances the selected sensory neural response and suppresses the irrelevant sensory neural response. With the additional visual perceptual load, more energy was diverted to the visual stimulus, which also led to AV suppression in addition to facilitation (Kamijo et al., 2007). Therefore, the AV depression effect under the medium and high visual perceptual-load conditions might be mainly attributed to the biased competition.

In addition, the response was slower with increasing visual perceptual load. Only one attribute (red color) was identified under low visual perceptual-load conditions; however, two attributes (red color and circle) were identified under medium visual perceptual-load conditions, and three attributes (red color, circle, double frame) were identified under high visual perceptual-load conditions. The capacity of the visual system for processing information is limited, and the performance was worse if the subject was instructed to identify two attributes than that to one attribute (Duncan, 1984; Kastner and Ungerleider, 2001). Therefore, the reduced response speed might be mainly attributed to the limited processing resources. Additionally, under the low visual perceptual-load conditions, the red color attribute is quickly and effortlessly detected, resulting from its salience, but it cannot be completed through the simple feature property under the medium and high visual perceptual-load conditions, which leads to a slower response. Therefore, another possible reason for the slower response with increasing visual perceptual load is the weakened salience for object identification than that for the simple feature.

Additionally, consistent with our original hypothesis, the AVI effect was higher in older adults than that in younger adults under low visual perceptual load conditions, which was also consistent with some of the previous studies reporting a higher AVI effect in older adults (Laurienti et al., 2006; Peiffer et al., 2007; Diederich et al., 2008; Deloss et al., 2013; Sekiyama et al., 2014). Although the primary brain regions for AVI are the same in older and younger adults, Grady (2009) reported that older adults recruited additional brain areas to participate in AVI (Grady, 2009). Diaconescu et al. (2013) used MEG to record the responses of subjects (15 younger adults and 16 elderly adults) to semantically related bimodal (V + A) and unimodal (V or A) sensory stimuli to capture the different brain areas involved in AVI. They reported that older individuals activated a specific brain network, the medial prefrontal cortex or posterior parietal cortex, when responding to audiovisual information. A comparative study between older and younger adults was also conducted by Ren et al. (2018) using an auditory/visual discrimination task, and they found activity in the visual cortex during AV stimuli processing in older but not in younger adults, which also indicated that older adults recruited the primary visual cortex involved in AVI. Additionally, reductions in hemispheric asymmetry in older adults have been found in many cognitive tasks, including episodic memory retrieval (Grady et al., 2002), perception (Grady et al., 1994, 2000), and working memory (Cabeza, 2002; Methqal et al., 2017). Therefore, the higher AVI effect of older adults, as a possible adaptive mechanism, might have mainly resulted from decreased brain specialization and activation of a distinct brain network to compensate for dysfunction in visual-only or auditory-only stimulus processing compared with younger adults (Gazzaniga et al., 2008; Koelewijn et al., 2010; Diaconescu et al., 2013; Baseler et al., 2014; Gutchess, 2014; Setti et al., 2014; Ren et al., 2018). However, the current studies could not identify whether the higher AVI is an adaptive mechanism or an indicator for unisensory decline, and future neuroimaging studies are necessary to clarify this matter. In addition, the AVI was significantly delayed in older adults compared with younger adults under all visual perceptual-load conditions, which was consistent with previous studies (Ren et al., 2018, 2020b). According to the “time-window-of-integration model” proposed by Diederich et al. (2008) before the integration of auditory and visual information (second stage), it is necessary to complete early auditory and visual information processing, which was assumed to be independent (first stage). There were significant sensory declines in older adults, showing slowed auditory and visual information processing in the first stage (Salthouse, 1996; Hirst et al., 2019); therefore, the delayed AVI with aging might be mainly attributed to age-related sensory decline.

## Conclusion

In conclusion, resulting from biased competition, the AVI effect was reduced with increasing visual perceptual load for both older and younger adults. It is possible that as a compensatory mechanism of the unimodal perceptual decline, the AVI of older adults was higher than that of younger adults, but their AVI effect was delayed resulting from age-related sensory decline.

## Data Availability Statement

The original contributions presented in the study are included in the article/Supplementary Material, further inquiries can be directed to the corresponding author/s.

## Ethics Statement

The studies involving human participants were reviewed and approved by the Ethics Committee of the Second Affiliated Hospital of Guizhou University of Traditional Chinese Medicine. The patients/participants provided their written informed consent to participate in this study.

## Author Contributions

YR and WY conceived and designed the experiments. YL and TW collected the data. YR analyzed the data and wrote the draft manuscript. HL revised the manuscript and responsed to the reviewers by receiving comments for WY. All authors contributed to the article and approved the submitted version.

## Conflict of Interest

The authors declare that the research was conducted in the absence of any commercial or financial relationships that could be construed as a potential conflict of interest.

## Publisher’s Note

All claims expressed in this article are solely those of the authors and do not necessarily represent those of their affiliated organizations, or those of the publisher, the editors and the reviewers. Any product that may be evaluated in this article, or claim that may be made by its manufacturer, is not guaranteed or endorsed by the publisher.

## Footnotes

1
http://stats.idre.ucla.edu/other/gpower/

2
https://audacity.onl/
